# Sheffield One‐Stop Service: A potential model to improve the screening uptake of diabetic peripheral neuropathy and other microvascular complications of diabetes

**DOI:** 10.1111/jdi.14268

**Published:** 2024-07-22

**Authors:** Gordon Sloan, Pepito Dela Pena, Aimee Andag‐Silva, Elaine Cunanan, Cecilia Jimeno, Jeremy Jones Robles, Solomon Tesfaye

**Affiliations:** ^1^ Division of Clinical Medicine University of Sheffield Sheffield UK; ^2^ Diabetes Research Unit Sheffield Teaching Hospitals NHS Foundation Trust Sheffield UK; ^3^ Section of Endocrinology, Diabetes and Metabolism East Avenue Medical Center Quezon City Philippines; ^4^ Section of Endocrinology and Diabetes De La Salle University Medical Center Cavite Philippines; ^5^ Section of Endocrinology, Diabetes and Metabolism University of St. Tomas Hospital Manila Philippines; ^6^ Section of Endocrinology, Diabetes and Metabolism University of the Philippines, Philippine General Hospital Manila Philippines; ^7^ Section of Endocrinology, Diabetes and Metabolism Chong Hua Hospital Cebu Philippines

**Keywords:** Diabetes Complications, Diabetic Neuropathy, Screening

## Abstract

The world is experiencing an enormous rise in the prevalence of diabetes, which is associated with massive healthcare costs that threaten to overwhelm many healthcare systems. Most of the diabetes expenditure is attributed to the management of chronic diabetes complications, including diabetic peripheral neuropathy (DPN)/diabetic foot complications, chronic kidney disease, sight‐threatening retinopathy and cardiovascular diseases. Of these complications, the most overlooked is DPN. Most consultations around the world do not even involve taking off shoes and socks to carry out a foot examination, and even when carried out, the peripheral neurological examination using the 10‐g monofilament diagnoses DPN when it is already at an advanced stage. Thus, all too often diabetes complications are diagnosed late, resulting in devastating outcomes, particularly in low‐ to middle‐income countries. There is, therefore, an urgent need to instigate new strategies to improve microvascular screening uptake using a holistic protocol for annual diabetes health checks outside the busy diabetes clinic. One such approach, the Sheffield One‐Stop Microvascular Screening Service, which involves modern point of care devices to diagnose DPN, has been shown to be feasible and effective, resulting in high uptake and early management of diabetes complications. This article outlines the advantages of this One‐Stop Microvascular Screening Service and a plan to trial an adapted version of this service to a resource‐limited country, the Philippines. If successful, this model has the potential for implementation in other countries around the world.

## Graphical abstract

Most of the diabetes expenditure is attributed to the management of chronic diabetes complications, including diabetic peripheral neuropathy/diabetic foot complications, chronic kidney disease, sight‐threatening retinopathy and cardiovascular diseases. There is, therefore, an urgent need to instigate new strategies to improve microvascular screening uptake using a holistic protocol for annual diabetes health checks outside the busy diabetes clinic. This article outlines the advantages of the Sheffield One‐Stop Microvascular Screening Service, a model that has the potential for implementation in other countries around the world.

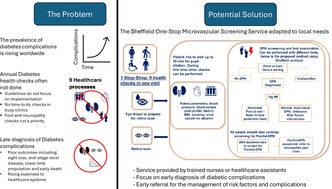

## INTRODUCTION

The worldwide prevalence of diabetes mellitus continues to rise at an unprecedented rate, up to 700 million people globally will be diagnosed with diabetes by 2045, an increase of one‐third from the current figure^1^. Although the prevalence is higher among high‐income countries (10.4%) compared with low‐income countries (4.0%), the greatest increase in the number of people with diabetes is expected to be in Africa, and 142.9% more people with diabetes are expected in 2045 compared with 2019. The rise in the prevalence of diabetes is largely driven by type 2 diabetes, due to adoption of the Western diet and lifestyle worldwide^2^. Diabetes and its complications impose an enormous economic burden on individuals, families and society. In 2021, the total estimated global healthcare expenditure for people with diabetes was nearly $1 trillion^3^. Although much of the economic burden related to diabetes is the direct costs related to healthcare, as much as 34.7% of global costs in 2015 were estimated to be indirect costs associated with loss of productivity predominantly due to labor‐force dropout. In addition, diabetes adversely impacts workforce productivity; for example, in 2010 there was an estimated $1 billion cost to the economy in Singapore alone due to lost productivity^4^. In Southeast Asian countries, where there is a high rate of personal ‘out‐of‐pocket’ healthcare expense, diabetes can impose catastrophic medical spending, leading to illness‐induced poverty^5^. Furthermore, as in other parts of the world, there is inequality in health in Southeast Asia, with poor access to appropriate diabetes care in lowest income and deprived communities^5^.

A major source of morbidity, mortality and healthcare expenditure in diabetes is the development of microvascular complications, namely retinopathy, nephropathy and peripheral neuropathy (DPN). DPN is a particularly neglected complication, which frequently is the precipitant for diabetic foot disease. In 2017, the global diabetic foot ulcer prevalence was 6.3% in people with diabetes^6^. The lifetime risk of diabetic foot ulceration has been reported to range between 12 and 25%, but more recent reports indicate the lifetime risk might be higher (19–34%) as a result of increases in life expectancy^7^, ^8^. Diabetic foot disease is associated with excessive morbidity, with recurrence rates of 65% at 3–5 years^8^, and lifetime risk of any lower‐extremity amputation in people with a diabetic foot ulceration is at least 19%^9^. Furthermore, the mortality of diabetic foot ulceration is shocking, at 50% at 5 years^10^, which is worse than many cancers, despite being perceived as less life‐threatening^11^.

The costs associated with diabetic foot disease are also enormous^12^. A detailed analysis from the UK between 2014 and 2015 showed that the cost of healthcare for ulceration and amputation was £837–962 million, 0.8–0.9% of the entire National Health Service budget for the UK^13^. Concerningly, these costs were calculated as 0.6% of the National Health Service budget between 2010 and 2011, suggesting a significant rise in the proportion of healthcare spending on diabetic foot^14^. The costs of diabetic foot care vary around the world, and depend on the healthcare system and economy. Furthermore, the data availability varies substantially too, and the health expenditure of diabetic foot care in low‐ and middle‐income countries is not well known^12^. In the USA, it is estimated that the cost related to diabetic foot ulceration amounts to $9–13 billion in excess of diabetes expenditure^15^. The disheartening expenses related to diabetic foot ulceration and amputations are projected to soar in the future with the growing population of diabetes globally^12^.

Despite screening for all these complications being recommended in international guidelines, the implementation of screening programs is scarce, particularly in low‐middle income countries. Only five out of 59 upper‐middle income counties have fully implemented diabetic eye‐screening programs, whereas no low‐/middle‐income countries do^16^. Whereas screening for chronic kidney disease in diabetes is approximately 86% in low‐to‐middle income countries^17^. The uptake of diabetic foot screening worldwide is unknown, due to a lack of data, but uptake in even high‐income countries, such as the UK, it is only 70–75%^18^. Among diabetes complications, DPN is often not a priority for busy physicians. The short diabetes consultation is focused on evaluation of blood glucose control, blood pressure, cholesterol and renal function. Many patients do not even have their shoes and socks taken off to examine the feet looking for DPN and peripheral arterial disease. Therefore, DPN is often not diagnosed at all, or diagnosed too late. Unfortunately, this results in well‐established neuropathy leading to diabetic foot ulceration, infection and amputations^19^. Even in well‐developed countries, up to 80% of patients with DPN remain undiagnosed^20^. Another recent study from four European countries showed that 50% of people with painful DPN also remain undiagnosed^21^. Thus, there is a need to develop screening programs for microvascular complications of diabetes, including DPN and painful DPN, which implement the recommendations of diabetes guidelines.

## DIABETIC PERIPHERAL NEUROPATHY

DPN is defined as a symmetrical, length‐dependent sensorimotor polyneuropathy that results from metabolic and microvascular alterations secondary to chronic hyperglycemia and other cardiovascular risk factors^22^. DPN is one of, if not the most, common chronic complications of diabetes. The global prevalence of clinically diagnosed DPN is estimated to be approximately 30% in people with diabetes, with a range of 6–51%^23^. However, when sensitive tests are used, such as nerve conduction studies, the reported prevalence is closer to 50%^24^, ^25^, ^26^.

The onset of DPN in type 1 diabetes patients generally occurs after several years, hence foot screening is only recommended 5 years after diagnosis^27^. However, DPN might be present in up to 13% of people with type 2 diabetes at diagnosis^28^, and can be present in people in prediabetic states, such as impaired fasting glucose and impaired glucose tolerance^29^. Indeed, the first presentation of diabetes can be with a diabetic foot complication, due to late diagnosis of diabetes and the development of already well‐established peripheral neuropathy.

The risk factors for DPN are well known, and include diabetes‐related factors, such as a longer duration of diabetes and a higher glycated hemoglobin (HbA1c; i.e., poorer diabetes control), and other potentially modifiable cardiovascular risk factors, such as dyslipidemia, obesity, hypertension and smoking^28^, ^30^. Hyperglycemia, dyslipidemia and other vascular factors lead to injurious molecular pathways within the whole nerve unit (neuron, Schwann cell, nerve vasculature, extra‐cellular matrix etc.), ultimately resulting in nerve cell death^31^.

In DPN, the longest nerves in the body are those that are impacted first, which are the nerves supplying the distal toes in both feet. DPN then progresses symmetrically to involve the feet and the legs in a stocking distribution. Once sensory loss reaches the level of approximately the knees, the upper limb can also be impacted. All nerve types are affected, including motor nerves, leading to foot deformity, and sudomotor nerves, which impair sweating in the feet, which can result in dried and cracked skin. Advanced DPN can also impair mobility and balance, leading to an increased risk of falling. DPN can be entirely asymptomatic, but some patients can experience symptoms, such as numbness, paresthesias (e.g., tingling, and pins and needles), and up to 50% suffer with neuropathic pain (Painful‐DPN). Painful neuropathic symptoms include burning, electric shock‐like and aching pain, and allodynia (pain due to a stimulus that does not normally provoke pain). Painful‐DPN often leads to severe pain, and can have a drastic impact on sleep, mood and quality of life^31^.

DPN is typically diagnosed clinically, using a combination of symptoms and careful clinical examination using simple instruments. Before attributing a neuropathy to DPN, a careful history and investigations should be carried out to exclude other potential causes of peripheral neuropathy (e.g., thyroid disease, vasculitis, vitamin B_12_ deficiency, alcohol neuropathy or uremia), whereas atypical features (e.g., absence of other microvascular complications, asymmetry of neurological impairment, rapid progression of symptoms or early involvement of the upper‐limb) might prompt referral to a specialist^31^.

The assessment of DPN has not changed in decades, and involves the use of crude and insensitive tools that are time consuming, and often not carried out by the busy physician in over‐stretched diabetes clinics. Assessment of temperature and pinprick (small‐fiber function), 128‐Hz tuning fork to assess vibration (large‐fiber function) and 10‐g monofilament test (foot ulcer risk) are minimum requirements according to guidelines; although, the Ipswich touch test can be carried out in resource‐limited countries, by lightly touching the tips of the first, third and fifth toes^27^, ^32^. However, these tests identify DPN at a late stage, when the underlying nerve injury is already advanced. The 10‐g monofilament in particular diagnoses DPN when there is a loss of protective sensation in the foot, and the foot is at risk of complications, such as ulceration. Progress has been made with the development of point‐of‐care devices that are capable of diagnosing DPN early, at a subclinical stage before it is clinically detectable^33^.

The devices with the most robust evidence are DPN‐Check and SUDOSCAN (Table 1)^34^, ^35^. DPN‐Check is a handheld device that measures sural nerve conduction velocity and amplitude in 3‐min. It has been shown to be a valid proxy for standard nerve conduction studies (95% sensitivity and 71% specificity)^35^, ^36^, which are expensive, time‐consuming and require neurophysiology experts to carry them out. SUDOSCAN provides a quantitative measurement of small fiber sudomotor function within 3‐min^33^. The device measures electrochemical skin conductance for the hands and feet, and has a sensitivity for classifying DPN of 87.5% and specificity of 76.2%^37^. Thus, these devices could be incorporated into new strategies to: (1) ensure that regular screening for DPN/foot ulcer risk are carried out, and (2) diagnose DPN at an earlier stage when the condition might be more amenable to treatment and to risk‐stratify patients.

**Table 1:** Clinical utility of DPN‐Check and SUDOSCAN for the diagnosis of diabetic peripheral neuropathy

	Function	Fibers assessed	Validated against	Sensitivity (%)	Specificity (%)	Intra‐observer intraclass correlation coefficient	Inter‐observer intraclass correlation coefficient
DPN‐Check	Sural sensory nerve function	Large Aα Aβ fibers	Nerve conduction studies, standardized clinical examination, and laser Doppler flare imaging	84.3–90.5%	68.3–86.1%	0.94–0.97	0.79–0.83
SUDOSCAN	Sudomotor function	Small C fibers	Nerve conduction studies, standardized clinical examination and thermal perception threshold	87.5%	87.5–76.2%	0.88	0.95

Adapted with permission by *Endocrine*, 2015^54^. Copyright and all rights reserved.

## INCREASING SCREENING UPTAKE FOR MICROVASCULAR COMPLICATIONS: THE SHEFFIELD ONE‐STOP SHOP

The International Diabetes Federation recommends all people with diabetes should have annual surveillance of diabetes control and complications, including assessment of blood glucose control (e.g., HbA1c), blood pressure, blood cholesterol, retinal screening, foot checks, urinary albumin and serum creatinine testing, weight, and smoking status check^38^. However, achievement of all these care processes is lacking, even in developed countries, such as the UK. Only 40% of people with type 1 diabetes and 50–60% with type 2 diabetes receive all these care processes in the UK, with little change in uptake over the past decade^18^.

The microvascular complication with the highest uptake is retinal screening, which has a nationally funded screening program that started in 2005. This screening program has led to a reduction in sight‐threatening retinopathy, such that diabetes is no longer the commonest cause of working‐age blindness, a phenomenon seen only in the UK, as opposed to the rest of the world^39^, ^40^, ^41^. In contrast, DPN diagnosis is overlooked, with the use of inappropriate screening instruments, such as the 10‐g monofilament test, which diagnoses the condition late.

**Figure 1:**
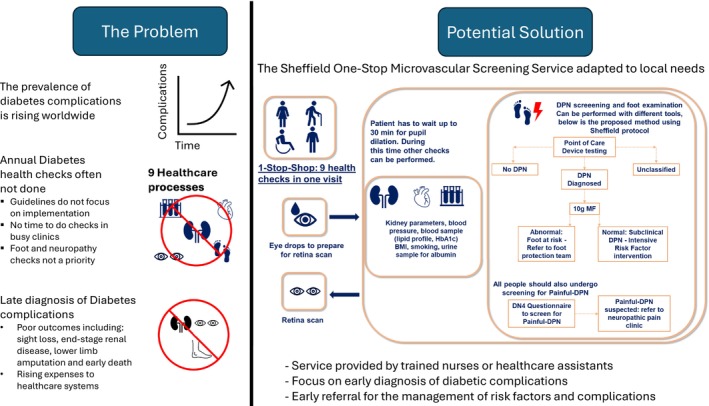
The Rationale for implementing the Sheffield One‐Stop Microvascular Screening Service in lower‐income countires. BMI, body mass index; DN4, Douleur Neuropathique en 4; DPN, diabetic peripheral neuropathy; HbA1c, glycated hemoglobin.

To tackle this poor screening uptake, the ‘Sheffield One‐Stop Microvascular Screening Service’ was developed^42^. The purpose of this service was to divorce the annual screening from the busy diabetes clinic and to have all the necessary care processes carried out by a nurse in one visit. Combining all checks in one visit ensures the screening and diagnosis of overlooked complications, such as DPN and painful‐DPN (Figure 1).

The Sheffield One‐Stop Service was developed through collaboration with the Sheffield Eye Screening Service to develop a comprehensive annual screening service^42^. On arrival, patients have mydriatic eye drops applied to dilate the pupils for digital retinal photography. This takes approximately 20 min, and this time is used to carry out anthropometric measurements (weight and waist‐hip ratio), blood pressure, blood tests (HbA1c, lipid profile, urea and electrolytes), urine test for urinary albumin excretion rate, smoking assessment and foot/neuropathy review. In addition, a novelty of this service is that it aims to diagnose DPN at an earlier stage, where it might be more amenable to treatment, and to risk stratify those at a high risk of foot disease and death, and to institute intensive risk factor treatment. To achieve this, the service utilizes point‐of‐care devices (DPN‐Check handheld device that measures nerve conduction within 3‐min and SUDOSCAN that assesses small fiber function within 5‐min) for the early diagnosis of DPN^34^, ^35^. However, in many resource‐limited countries, these devices will not be available, and DPN screening can be carried out using other widely available diagnostic tools, such as the 128‐Hz tuning fork (large‐fiber function) and Neurotips (small‐fiber function), and even in remote areas with extremely poor resources, such as the Ipswich Touch‐Test^27^, ^32^. Furthermore, a simple and quick screening tool for neuropathic pain is used, the Douleur Neuropathique en 4 (DN4), to diagnose painful‐DPN^43^. Finally, the patients have retinal photography, with the whole process taking approximately 30 min. The advantage of this approach is that the whole process can be carried out by trained nurses or allied healthcare professionals.

Altogether, data from 236 participants were analyzed (mean age 63.5 years, standard deviation 14.1 years; 97.8% type 2 diabetes; 61.4% men)^42^. Only 18.9% had documented evidence of foot screening within the previous 12 months. The prevalence of DPN was underestimated by the 10‐g monofilament test (14.4%) compared with an abnormality in either DPN‐Check or SUDOSCAN (61.9%), which had a sensitivity of 93.2% to detect DPN. Furthermore, using our proposed algorithm (Figure 1), 29% would have DPN excluded (sensitivity 95%), 36% had DPN diagnosed (specificity 82.2%) and 35% were ‘unclassified’.

We propose that those without DPN could undergo biennial screening, and those unclassified undergo screening yearly; whereas those with DPN with a positive 10‐g monofilament would be referred to a foot protection team, and those with a negative 10‐g monofilament (subclinical DPN) could undergo intensive risk factor intervention. The study also diagnosed 25% of participants with painful‐DPN. Furthermore, 91.1% of the participants indicated they were ‘in favor’ or ‘strongly in favor’ of the service, with no‐one against the service.

Thus, the Sheffield One‐Stop Microvascular Screening Service was shown to have a high uptake, reducing clinic visits, and identified early DPN and Painful‐DPN^42^. The service allows the opportunity to provide patients with their risk factors and complication status (in a patient‐friendly information leaflet) in one visit, so people can be empowered to be drivers of their own treatment. There is a potential to develop the information leaflet into a mobile phone application, which could track risk factor/complication status over time and provide advice on accessing treatment locally. A robust business case for the cost‐effectiveness of the service was developed, and following engagement with the Sheffield Care Commissioning Group, this model of care was accepted and is in operation in four very large general practice hubs in the city, which has an estimated 40 000 people with diabetes (Table 1).

## ADAPTING THE ONE‐STOP MICROVASCULAR SCREENING SERVICE MODEL TO OTHER RESOURCE‐LIMITED COUNTRIES: A CASE STUDY IN THE PHILIPPINES

The Philippines is in the Western Pacific Ocean part of Southeast Asia, with a population of approximately 113.9 million. It has a high incidence of poverty (i.e., proportion of people falling below the national poverty line) of approximately 18.1%, the third highest in Southeast Asia^44^. Adoption of the Western lifestyle has led to a rise in the prevalence of elevated fasting blood sugar suggestive of diabetes, from 3.4% in 2003 to 7.9% in 2018^45^. The Philippines also has one of the highest rates of undiagnosed diabetes in the world at 66.7%, second only to Indonesia in the region^46^. Diabetes ranks as the fourth commonest cause of death within the country^47^, and microvascular complications are prevalent^48^, ^49^. Although robust data for diabetic complications are not available, the rates of amputations and end‐stage renal disease are high^50^, ^51^, ^52^. Although national guidelines for the management of diabetes and microvascular disease screening are available, effective implementation of the guidelines is lacking. This results in late and irreversible sequelae of diabetes, high mortality, and healthcare costs with a two‐ to fourfold increase in the expenses seen among those with complications versus those without, based on a recent local study^53^.

The high rates of diabetes complications in the Philippines, where physicians have little time to do comprehensive screening in busy clinics, led to a meeting with endocrinologists from the Philippines and Sheffield at the International Diabetes Federation conference in Lisbon 2022, where a decision was made to explore adaption of the Sheffield One‐Stop Microvascular Screening Service to the healthcare system in the Philippines. A meeting of key stakeholders (endocrinologists, diabetes guideline developers, ophthalmologists, hospital health managers, and healthcare research board members of the Philippines College of Endocrinology, Diabetes and Metabolism) occurred in 2023, exploring the challenges and opportunities of adapting the One‐Stop Shop to the specific healthcare system in the Philippines. A 6‐month feasibility study within East Avenue Medical Center in Manila will take place, which will carry out an adapted One‐Stop‐Shop clinic for 300 patients attending for annual diabetes reviews. Patients will have mydriatic eye drops applied to dilate pupils for digital retinal photography/direct fundus examination; while awaiting mydriasis, they will undergo other care processes carried out by a well‐trained nurse, including foot/neuropathy screening; DN4 questionnaire to diagnose painful‐DPN; anthropometric measurement (weight and blood pressure); and biochemical testing (serum HbA1c, lipid profile, and renal function and urine albumin creatinine ratio). Finally, retinal assessment will occur within the ophthalmology department. This project will determine the practicality of adapting the Sheffield One‐Stop Microvascular Screening Service in the healthcare settings in the Philippines, and allow a detailed cost analysis of the service.

## CONCLUSIONS

It is well recognized that most people with diabetes do not have appropriate screening services, especially in low‐ and middle‐income countries. Although there are high‐quality clinical guidelines for the management of diabetes, their implementation in clinical practice, as in many parts of the world, is woefully inadequate. In the UK, the institution of an annual digital camera‐based eye screening since 2005 for all people with diabetes has led to displacing diabetic retinopathy as the leading cause of working‐age blindness. This national screening has very high uptake (>85%), and patients are referred at the earliest changes to an ophthalmologist. This service has been a game‐changer. A similar approach of robust, cost‐effective screening services is required if a significant reduction in late complications is to be achieved. In particular, the One‐Stop comprehensive screening will ensure the early diagnosis and management of currently neglected complications, such as DPN and Painful‐DPN.

This feasibility study of starting the One‐Stop Shop primarily carried out by well‐trained nurses outside the diabetes clinic with the results fed to their physician is, therefore, the first step in the process. Depending on resource availability, the use of objective and quantitative measures of DPN using point‐of‐care devices will ensure early diagnosis. If there is good feasibility of conducting this One‐Stop Shop, both in hospital and community settings, further studies will be required to see if such services can be offered in large primary care hubs, as in Sheffield, the UK. This will require the engagement and active participation of patient groups, healthcare providers, policy makers, guideline writers and politicians working in concert to embrace this model, and invest in its infrastructure and ensure sustainability. If the results of the feasibility study within the Philippines are favorable, this will be the first step for further studies aimed at implementing such a service both in community and hospital settings. The project carried out in the Philippines will be a pilot showing the feasibility in similar healthcare settings in other countries and can be reapplied.

## DISCLOSURE

GS has received honoraria from Procter & Gamble. AS has received honoraria from Proctor & Gamble, and CME grants from Cathay YSS, UniLab, Natrapharm and Getz Pharma. PD has been on the advisory board for GX Pharma and Procter & Gamble. EC has been speaker for Medichem Pregabalin Treasurer, Philippine College of Endocrinology, Diabetes and Metabolism. ST has received: honoraria from Procter & Gamble, Viatris, Grunenthal, Novo Nordisk, Merck, Eva Pharma, Hikma, Astellas Pharma, Abbott, AstraZeneca, Berlin‐Chemie, Worwag Pharma and Nevro, Haisco Pharmaceutical Group for educational meetings; consultancy fees for advisory board membership from Angelini, Bayer, Worwag Pharma and Nevro; research equipment donated to Sheffield Teaching Hospitals from Impeto Medical and Neurometrix; and unrestricted, competitive research grants from Viatris and Procter & Gamble.
